# Statistical analysis plan for a randomized controlled trial examining pedometer-based walking intervention in patients with heart failure with reduced ejection fraction: the WATCHFUL trial

**DOI:** 10.1186/s13063-023-07516-5

**Published:** 2023-08-17

**Authors:** Tomas Vetrovsky, Michal Siranec, Tereza Frybova, Iulian Gant, Miroslav Semerad, Marie Miklikova, Vaclav Bunc, Jiri Vesely, Jiri Stastny, Martin Griva, Jan Precek, Radek Pelouch, Jiri Parenica, Jiri Jarkovsky, Jan Belohlavek

**Affiliations:** 1 Faculty of Physical Education and Sport, Charles University, Prague, Czech Republic; 2 2nd Department of Medicine – Department of Cardiovascular Medicine, First Faculty of Medicine, Charles University and General University Hospital in Prague, Prague, Czech Republic; 3 Cardiology Department, University Hospital Brno and Medical Faculty of the Masaryk University, Brno, Czech Republic; 4 Faculty of Medicine in Hradec Kralove, Charles University, Prague, Czech Republic; 5 Edumed S.R.O, Broumov, Czech Republic; 6 Department of Cardiology, Tomas Bata Regional Hospital, Zlin, Czech Republic; 7 Innere Medizin I - Abteilung Für Kardiologie Und Internistische Intensivmedizin, Landesklinikum Mistelbach, Mistelbach, Austria; 8 Department of Internal Medicine I - Cardiology, University Hospital Olomouc, Olomouc, Czech Republic; 9 1st Department of Internal Medicine – Cardioangiology, Faculty of Medicine in Hradec Kralove, Charles University, Prague, Czech Republic; 10 University Hospital Hradec Kralove, Hradec Králové, Czech Republic; 11 Institute of Biostatistics and Analyses, Faculty of Medicine, Masaryk University, Brno, Czech Republic

**Keywords:** Physical activity, Phone counseling, Tele-rehabilitation, Functional capacity, Six-minute walk test, Self-monitoring, Wearables, Garmin, Pragmatic trial, Intention-to-treat analysis

## Abstract

**Background:**

Physical activity is an effective management strategy for heart failure with reduced ejection fraction, but patients’ compliance is challenging. Walking is a suitable form of physical activity due to its convenience and sustainability, and it can potentially improve functional capacity in heart failure patients.

**Objectives:**

The WATCHFUL trial aims to determine whether a pedometer-based walking intervention combined with face-to-face sessions and regular telephone contact improves functional capacity in heart failure patients.

**Methods:**

The WATCHFUL trial is a 6-month multicenter, parallel-group, randomized, controlled, superiority trial with a 6-month follow-up. A total of 202 patients were recruited for the trial. The primary analysis will evaluate the change in distance walked during the 6-min walk test from baseline to 6 months based on the intention-to-treat population; the analysis will be performed using a linear mixed-effect model adjusted for baseline values. Missing data will be imputed using multiple imputations, and the impact of missing data will be assessed using a sensitivity analysis. Adverse events are monitored and recorded throughout the trial period.

**Discussion:**

The trial has been designed as a pragmatic trial with a scalable intervention that could be easily translated into routine clinical care. The trial has been affected by the COVID-19 pandemic, which slowed patients’ recruitment and impacted their physical activity patterns.

**Conclusions:**

The present publication provides details of the planned statistical analyses for the WATCHFUL trial to reduce the risks of reporting bias and erroneous data-driven results.

**Trial registration:**

ClinicalTrials.gov (identifier: NCT03041610, registered: 3/2/2017).

**Supplementary Information:**

The online version contains supplementary material available at 10.1186/s13063-023-07516-5.

## Background

Heart failure with reduced ejection fraction (HFrEF) is a debilitating condition affecting millions worldwide [1]. Patients with HFrEF often experience impaired functional capacity, reduced quality of life, and increased risk of hospitalization and mortality [2]. In recent years, there has been growing evidence suggesting that physical activity plays a crucial role in the management of HFrEF [3, 4]. Despite the numerous benefits of physical activity for patients with HFrEF, many patients struggle to comply with physical activity recommendations [5, 6]. Studies have consistently shown that patients with HFrEF have low physical activity levels, often falling short of recommended guidelines [7].

Walking can be an especially suitable form of physical activity for patients with HFrEF: it is a low-impact, weight-bearing exercise that can improve patients’ functional capacity and reduce their symptoms [8]. Walking is easy to perform and requires no special equipment, making it a convenient and sustainable option for most patients. Simple strategies such as brief advice during a face-to-face session with a physician, setting a step goal and self-monitoring the progress with a pedometer, and regular phone support from healthcare providers have the potential to improve the walking levels of HFrEF patients [9–11]. These strategies can be tailored to the individual needs and abilities of each patient and can be integrated into routine clinical care to help patients improve their functional capacity, ultimately leading to better outcomes in the management of HFrEF [12].

Despite this potential, delivering evidence-based walking interventions for HFrEF patients in clinical settings is not common practice [13]. One possible explanation is that many interventions are designed to achieve maximum effect in explanatory trials but are difficult to translate into routine clinical practice and fail when moved along the translation continuum [14]. Thus, effective and sustainable walking interventions for HFrEF patients that can be translated into routine clinical care are lacking and urgently needed.

We have designed the WATCHFUL randomized controlled trial with the aim to determine whether a 6-month pedometer-based walking intervention combining face-to-face sessions and regular telephone contact improves functional capacity assessed by the 6-min walk test (6MWT) in patients with HFrEF compared to usual care [15]. The trial has been designed as a pragmatic trial to ensure that the intervention, if shown to be effective, can be translated into routine clinical practice.

## Objectives

The WATCHFUL trial design has been described in detail in the published trial protocol, which also included a brief overview of the statistical analyses [15]. However, primary statistical analyses should be pre-specified in detail to prevent the data-driven choice of analyses and selective reporting of outcomes [16, 17]. Thus, this article presents the detailed statistical analysis plan (version 1.0), which was finalized in May 2023, prior to the final participant follow-up, and follows the Guidelines for the Content of Statistical Analysis Plans in Clinical Trials [18].

## Methods

### Trial design

The WATCHFUL trial is a 6-month multicenter, parallel-group, randomized, controlled, superiority trial with a 6-month follow-up. The trial protocol has been approved by the Ethics Committee of the General University Hospital, Prague (20/16 Grant VES 2017 AZV VFN), and the trial has been registered in ClinicalTrials.gov (identifier: NCT03041610, registered: 3/2/2017, https://clinicaltrials.gov/ct2/show/NCT03041610).

The trial recruited patients with HFrEF from six cardiovascular centers, five of them in academic hospitals throughout the Czech Republic. Patient recruitment started in August 2018 and was completed in December 2022; thus, the 6-month intervention is anticipated to finish in June 2023, and the 6-month follow-up in December 2023. Compared to the timelines reported in the published trial protocol, both the start and end of the trial were delayed: the start was delayed by 16 months due to a lack of funding, and the end was delayed by additional 20 months due to the COVID-19 pandemic that slowed down the recruitment. The primary analysis will be performed after the intervention phase at six months. No interim analyses have been planned before the end of the intervention phase.

### Eligibility

Patients with HFrEF (left ventricular ejection fraction < 40%) and New York Heart Association (NYHA) class II or III symptoms aged at least 18 years were eligible for the study. In contrast to the published study protocol, physical inactivity (determined by asking the patient a single-item question) was not considered an eligibility criterion.

The exclusion criteria were as follows: (1) signs and symptoms of decompensated heart failure, uncontrolled arrhythmia or effort angina, severe or symptomatic aortic stenosis, or persistent hypotension; (2) recent (< 3 months) myocardial infarction, percutaneous coronary intervention, implantation of an implantable cardioverter defibrillator or bi-ventricular pacemaker, or shocks delivered by the automated implantable cardioverter defibrillator; (3) co-morbid conditions potentially affecting adherence to trial procedures (e.g., inflammatory arthritis, active malignancy, a renal disease requiring dialysis, uncontrolled diabetes, major depression or other significant psychiatric disorders, cognitive impairment, or significant hearing or visual impairment); (4) major surgery planned within the next 12 months; (5) life expectancy shorter than 12 months; (6) inability to walk for any reason; (7) pregnancy; (8) baseline 6-min walking distance > 450 m; and (9) failure to perform the 6MWT.

### Sample size

For the purpose of the power analysis, we chose a change in six-minute walk distance of 45 m, which is considered the minimum clinically important difference in patients with heart failure [19]. The standard deviation of the response variable in similar populations varies between 38 and 96 m [19]. Therefore, to detect a clinically meaningful change of 45 m on the 6MWT with a power of 80% using a 2-sided 0.05 significance level (alfa) and assuming that the standard deviation is 100 m, 79 participants in each group are needed. To account for an expected attrition rate of 20%, we planned to recruit 100 patients for each group, resulting in 200 patients in total. Ultimately, due to challenges in coordinating recruitment efforts across the six different participating centers, 202 patients were recruited into the trial, with each group consisting of 101 patients.

### Screening and recruitment

Participants were identified during routine clinical visits at each of the participating centers. Potential participants (i.e., patients with HFrEF and NYHA class II or III symptoms, aged at least 18 years, with the absence of exclusion criteria 1 to 7 as listed in the Eligibility section) underwent a screening phase, which consisted of the 6MWT. Participants who walked more than 450 m during the 6MWT (exclusion criterion 8) or those who failed to perform the 6MWT (exclusion criterion 9) were excluded.

The total number of HFrEF patients evaluated for the absence of exclusion criteria 1 to 7 (i.e., the criteria that can be assessed based on electronic medical records and routine examination) was not collected during this study as it would not be sufficiently reliable. However, the number and characteristics of the patients who underwent the screening phase (i.e., the 6MWT) were recorded and will be reported.

Following the screening phase, a physician or a research nurse explained the study in detail to all eligible patients. Those who agreed to participate were provided with an informed consent form, indicating their full understanding of the study and their protected rights for confidentiality and withdrawal from the study without giving a reason.

### Randomization and blinding

After their consent, the patients were randomly assigned in a 1:1 ratio to either the intervention or control group. The randomization was performed centrally, using a computer-automated randomization system to guarantee adequate allocation concealment. The trial used a permuted block randomization scheme stratified by center, NYHA class, sex, and age (18–65, ≥ 66) to ensure equal representation in the groups.

Due to the nature of the trial protocol, the patients and researchers could not be blinded, as they are both aware of the allocation due to their active role in the intervention. However, all assessments are undertaken by assessors blinded to treatment allocation.

### Intervention and control groups

Patients allocated to the intervention group receive a 6-month intervention consisting of an individualized pedometer-based walking program with weekly step goals, face-to-face sessions with the physician, and regular telephone calls with the research nurse in between the face-to-face contacts. At baseline, the patients received a wrist-worn pedometer Garmin vívofit that has been previously validated in heart failure patients [20] and were instructed to self-monitor their daily step count and increase it gradually by at least 3000 steps over their baseline steps. At baseline and 3 and 6 months, the patients visit their physician, who encourages them to integrate walking into their daily routine and reminds them of the health benefits of walking. In addition, the patients receive regular monthly phone calls from one of two research nurses who assesses patients’ progress, provides individualized feedback, monitors their adherence, discusses their personal goals, assists them in identifying barriers and solutions to physical activity participation, and provides encouragement.

Patients allocated to the control group receive their usual care. At the baseline visit, they were educated about the beneficial effects of regular physical activity and encouraged to increase their physical activity level; however, they have not received the pedometer and regular phone calls from the research nurse.

### Progress through the trial

The progress of all participants through the trial, beginning with the screening phase and including all time points until the follow-up at 12 months, will be summarized and reported in a CONSORT flow diagram [21]. Timing and the level of consent withdrawal will be presented by a trial group within the CONSORT flow diagram. The level of consent withdrawal will be classified as either withdrawal from the intervention, withdrawal from follow-up, or complete withdrawal from the trial. The investigators will make a reasonable effort to ascertain the reasons for withdrawal and summarize the reasons by a trial group. Losses to follow-up and their reasons will be tabulated and reported by a trial group for the individual time points.

## Statistical analysis

### Baseline patient characteristics

Baseline patient characteristics will be presented, both overall and separately for the two randomized groups, and will include age, sex, marital status, education level, employment status, smoking status, alcohol intake, average daily step count, distance walked during the 6MWT, blood pressure, body mass index, waist circumference, NYHA class, ejection fraction, N-terminal pro–B-type natriuretic peptide (NT-proBNP), high-sensitivity C-reactive protein (hsCRP), creatinine, heart failure history and etiology, comorbidities, current medication, and Meta-Analysis Global Group in Chronic heart failure (MAGGIC) risk score [22].

To assess whether attrition has introduced selection bias or upset the balance achieved at randomization, baseline characteristics will also be presented for the analysis population included in the primary analysis of the primary outcome.

Categorical data will be summarized by numbers and percentages. Continuous data will be summarized by mean and SD if data are normal and by median, interquartile range, and 5th and 95th percentiles if data are skewed. Tests of statistical significance will not be undertaken for baseline characteristics.

### Adherence and protocol deviations

Adherence to the intervention will be assessed based on the percent of subjects who either (a) have refused to wear the wrist-worn activity tracker, (b) have not shown up for the clinic visit at 3 and 6 months, or (c) have not engaged in at least 3 out of 5 planned phone counseling sessions. Descriptive statistics on the percent adherence will be summarized for the intervention group.

The following are pre-defined major protocol violations with a direct bearing on the primary outcome: (1) failure to perform the baseline 6MWT in enrolled participants. The baseline 6MWT is conducted after patients’ enrollment (i.e., after obtaining their informed consent) to screen for their eligibility (patients covering more than 450 m in the baseline 6MWT are excluded according to the protocol); (2) failure to randomize enrolled and eligible participants; (3) failure to provide intervention to participants allocated to the intervention group, specifically, not providing them with the pedometer or not encouraging them to increase their daily step count; (4) erroneous provision of the intervention to the control group; (5) failure to complete the assessment of the primary outcome at 6 months.

The pre-defined minor protocol violations include but are not limited to (1) enrollment of non-eligible participants; (2) variations in the timing of the intervention or assessments; (3) non-adherence to the intervention as defined above; and (4) non-compliance with data collection procedures.

The number (and percentage) of patients with major and minor protocol deviations will be summarized by treatment group with details of the type of deviation provided. The number of patients included in the intention-to-treat analysis data set will be used as the denominator to calculate the percentages. No formal statistical testing will be undertaken.

### Analysis population

The intention-to-treat population will include all randomized patients, regardless of their eligibility, according to the treatment they were randomized to receive.

The per-protocol analysis set will consist of subjects who (1) were randomly assigned to treatment, (2) have no major protocol violations, (3) comply with the eligibility criteria, (4) have both a baseline and 6-month measurement on the given outcome with the variation in the timing of the 6-month measurement not greater than one month, (5) adhered to the intervention (see the “Adherence and protocol deviations” section for the adherence criteria), and (6) complied with the data collection procedures for the given outcome.

### Outcome definitions

The primary outcome is the change in distance (in meters) walked during the 6MWT from baseline to 6 months. The secondary outcomes and time points of their measurement are summarized in Table 1 and detailed in the published trial protocol [15]. We deviated from the protocol by not evaluating lung ultrasound due to logistical constraints and limited resources.

**Table 1:** Overview of the study outcomes

Outcome	Baseline	3 months	6 months	12 months
Sociodemographic characteristics, medical history	✓			
Clinical examination (NYHA class, vital signs)	✓	✓	✓	✓
Six-minute walk test	✓	✓	✓	✓
Echocardiography (ejection fraction)	✓		✓	✓
NT-proBNP	✓	✓	✓	✓
hsCRP	✓		✓	✓
Physical activity measured by Actigraph	✓		✓	✓
Beck depression inventory-II (BDI-II)	✓		✓	✓
36-item short-form health survey (SF-36)	✓		✓	✓
General self-efficacy scale (GSE)	✓		✓	✓
Body weight, height	✓	✓	✓	✓
Waist and hip circumference	✓		✓	✓
MAGGIC risk score	✓		✓	✓
Adverse events	✓	✓	✓	✓

The average daily step count is measured by the hip-worn Actigraph wGT3X-BT accelerometer over a 7-day period and calculated using a proprietary algorithm within the ActiLife software. The non-wear time is detected using the Choi algorithm as implemented in the ActiLife software. In contrast to the trial protocol but in line with the latest recommendations [23], the valid day is defined as at least 600 min of wear time, and only measurements with at least four valid days, including at least one weekend day, will be included in the analyses.

### Analysis methods

The primary analysis will evaluate the change in distance walked during the 6MWT from baseline to 6 months based on the intention-to-treat population. The analysis will be performed using a linear mixed-effect model accounting for clustering at the center level (random effect) and adjusted for baseline distance walked during the 6MWT, age, sex, and NYHA class (fixed effects). The intervention effect (adjusted value for change in intervention minus change in control) will be reported as the mean (95% CI) and associated *p*-value. Assumptions of normality will be tested, and an alternative distribution will be used where necessary. A sensitivity analysis on the per-protocol population will be performed to test the robustness of the primary analysis. All applicable statistical tests will be 2-sided and performed using a 5% significance level.

The secondary analyses will evaluate changes in the 6MWT distance from the baseline to 3- and 12-month time points and changes in secondary outcomes from the baseline to all available time points (Table 1). The secondary analyses will be performed using the same approach as the primary analysis. Individual *p*-values will not be reported for secondary analyses as the adjustment for multiple testing will not be undertaken. However, we will present two-sided 95% confidence intervals for all outcomes to allow readers to make their own interpretations of the results.

Several exploratory subgroup analyses are planned to assess the differential effect of the intervention on patients (1) participating before, during, and after the COVID-19 pandemic and (2) allocated to either of the two research nurses providing phone support.

### Missing data

Missing data will be imputed using multiple imputations created by predictive models based on the patients with complete data [24], and the impact of missing data will be assessed using a sensitivity analysis.

### Harms

Adverse events are monitored and recorded throughout the trial period. Data regarding falls, injuries, musculoskeletal problems, major cardiovascular disease events, and any other events potentially related to the implementation of the trial protocol are collected at each time point. The number and percentage of occurrences of each adverse event will be presented for each trial group separately. No formal statistical testing will be undertaken.

### Statistical software

All analyses will be carried out using R statistical software. The packages and their version numbers used for analyses will be recorded and reported.

## Discussion

### Strengths

Our methods have several important strengths. First, the trial has been designed as a pragmatic trial to ensure that if shown to be beneficial, the intervention could be translated into routine clinical care.

Second, we designed the intervention as scalable so that it could be easily rolled out to routine clinical practice without incurring prohibitive costs or placing an additional burden on the medical staff in order to facilitate trial translatability.

Third, the conclusions will be principally based on the results of the primary outcome. For secondary outcomes, we will present two-sided 95% confidence intervals but not report *p*-values, thereby limiting problems of multiplicity.

Fourth, the primary outcome, the distance walked during the 6MWT, is a patient-centered outcome closely associated with patients’ functional status. Additionally, the 6MWT is commonly used in the management of HFrEF patients, making it relatively simple for clinicians to interpret.

Finally, the intention-to-treat principle of the primary analysis will provide an unbiased estimate of the intervention’s effectiveness, maintain the randomization of study groups, prevent potential biases from selective exclusion, and provide a conservative estimate of the intervention effect.

### Limitations

The trial also has several limitations. We recorded the number and characteristics of the patients who underwent the screening phase using the 6MWT. However, due to practical reasons, we could not record the number and characteristics of all patients who were considered for inclusion but did not enter the screening phase due to the presence of exclusion criteria 1 to 7. This limits the external generalizability of the trial.

Another potential limitation is that phone support for all intervention patients is provided by only two research nurses who are closely associated with the research team and highly motivated. As the intervention impact likely depends on the quality of the phone support, the intervention effect can be reduced when the intervention is translated to routine clinical care, and nurses of various qualities and motivations will be recruited to provide the phone support. To investigate the potential impact of the varying phone support, we plan to conduct an exploratory analysis comparing patients allocated to either of the two research nurses.

Finally, the trial was substantially affected by the COVID-19 pandemic, which had a significant impact on patients’ habitual physical activity [25] and contributed to the serious delay in patients’ recruitment. To assess the differential effect of the intervention on patients participating before, during, and after the COVID-19 pandemic, we plan to conduct an exploratory analysis of the respective patient subgroups.

## Conclusions

The present publication provides details of the planned statistical analyses for the WATCHFUL trial to reduce the risks of reporting bias and erroneous data-driven results.

### Supplementary Information

**Additional file 1.** Statistical Analysis Plan Checklist.

## Data Availability

Not applicable.

## Abbreviations

HFrEF: Heart failure with reduced ejection fraction
6MWT: Six-minute walk test
NYHA: New York Heart Association
hsCRP: High-sensitivity C-reactive protein
NT-proBNP: N-terminal pro–B-type natriuretic peptide
MAGGIC: Meta-Analysis Global Group in Chronic heart failure
